# Beverage-based caffeine intake and high consumption trends among Korean adults, 2016–2024: a nationwide population-based study

**DOI:** 10.3389/fnut.2026.1913422

**Published:** 2026-10-19

**Authors:** Jinmi Kim, Gyu Lee Kim, Young Jin Tak, Seung Hun Lee, Young Jin Ra, Yu Hyeon Yi

**Affiliations:** 1 Department of Biostatistics, Clinical Trial Center, Biomedical Research Institute, Pusan National University Hospital, Busan, Republic of Korea; 2 Department of Family Medicine and Medical Research Institute, Pusan National University Hospital, Busan, Republic of Korea; 3 Department of Family Medicine, Pusan National University School of Medicine, Yangsan, Republic of Korea

**Keywords:** beverage consumption, caffeine intake, coffee consumption, Korea National Health and Nutrition Examination Survey (KNHANES), temporal trends

## Abstract

**Background:**

Caffeine is the most widely consumed psychoactive substance worldwide, yet nationally representative evidence on long-term consumption trends remains limited in Korea. This study aimed to evaluate temporal trends in beverage-derived caffeine intake among Korean adults between 2016 and 2024, identify major beverage sources, and examine demographic factors associated with high caffeine intake (≥400 mg/day).

**Methods:**

Data were obtained from the 7–9th Korea National Health and Nutrition Examination Surveys. We included adults aged ≥19 years with valid 24-h dietary recall data. Caffeinated beverages were identified using beverage food codes and product information recorded in the dietary database and classified into five categories: coffee, tea, energy drinks, cola-type beverages, and cocoa-based beverages. Daily caffeine intake was estimated using beverage-specific caffeine concentrations derived from national monitoring data. Survey-weighted linear and logistic regression models accounting for the complex sampling design were used to assess temporal trends and associations with high caffeine intake.

**Results:**

Among 48,991 adults, mean daily caffeine intake increased over time (β = 1.66 mg/day per year; 95% CI, 1.05–2.27), and the odds of high caffeine intake also increased (OR = 1.05 per year; 95% CI, 1.02–1.07). Coffee was consumed by 90.8% of caffeine consumers, with a shift from coffee mixes toward brewed coffee. Women aged 30–44 years had higher odds of high caffeine intake (aOR = 1.62; 95% CI, 1.21–2.16), whereas adults aged ≥65 years had lower overall odds (men: aOR = 0.37; 95% CI, 0.25–0.53; women: aOR = 0.23; 95% CI, 0.12–0.44).

**Conclusions:**

Caffeine intake among Korean adults has increased, driven by shifts in coffee consumption. The rising odds of high caffeine intake among working-age adults highlights the need for continued surveillance and targeted public health guidance.

## Introduction

Caffeine is the most commonly consumed psychoactive compound worldwide and is mainly obtained from beverages, including coffee, tea, carbonated drinks, and energy drinks (1). While moderate caffeine consumption leads to enhanced alertness and cognitive functioning, excessive consumption is associated with undesirable effects such as sleep disruption, anxiety symptoms, and increased cardiovascular burden (2–4).

Previous investigations have explored relationships between coffee or caffeine intake and various health outcomes, including metabolic disorders, obesity, hypertension, and indicators of mental health in Korea (5, 6). Recent prospective evidence has also suggested that moderate consumption of caffeinated coffee and tea may be associated with a lower risk of dementia and better cognitive outcomes (7). Higher caffeine intake also co-occurs with other lifestyle characteristics, such as smoking, alcohol use, and occupational stress (8, 9). Much of the literature is limited to analyses of single survey cycles or selected population groups, including students or younger adults (10, 11). Consequently, comprehensive evidence regarding long-term temporal trends in caffeine intake in the general adult population, shifts in the upper tail of intake distribution, beverage-specific sources of caffeine, and factors associated with high intake levels, particularly consumption >400 mg per day (upper reference level for adults) remains scarce (11, 12).

Estimating caffeine intake based on 24-h dietary recall data involves several methodological considerations (13). Without systematic harmonization, these sources of variability may introduce exposure misclassification (14). Nonetheless, the 24-h dietary recall approach remains suitable for evaluating short-term intake of nutrients characterized by substantial day-to-day variation, such as caffeine, and enables item-level estimation within a nationally representative sample (15, 16).

Given ongoing changes in the beverage environment and increasing public interest in the health implications of caffeine (17, 18), developing a standardized analytical framework is essential to enhance comparability across survey years and to inform evidence-based dietary recommendations.

Accordingly, we assessed beverage-derived caffeine intake among Korean adults aged ≥19 years using KNHANES data collected between 2016 and 2024. We investigated long-term trends in total caffeine intake, changes in intake distribution, and contributions by beverage type, with particular emphasis on high intake levels (≥400 mg/day). Additionally, we implemented a standardized strategy for food code harmonization and caffeine estimation to reduce coding-related inconsistencies across survey cycles. By generating robust national estimates and presenting a reproducible methodological approach, we aimed to strengthen nutrition surveillance and support public health strategies related to caffeine consumption in Korea.

## Methods

### Study population and data source

Data were obtained from the 7–9th Korea National Health and Nutrition Examination Survey (KNHANES, 2016–2024), a nationally representative survey conducted using a complex multistage probability sampling design. Adults aged ≥19 years with valid 24-h dietary recall data were included in the analysis. This study was approved by the Institutional Review Board of Pusan National University Hospital (IRB No. 2602-019-160), and the requirement for informed consent was waived because this study used anonymized secondary data from KNHANES.

### Identification of caffeinated beverages

Dietary intake data were obtained from the 24-h dietary recall survey. Beverage records were initially identified using dish codes corresponding to the “beverages” and “teas/coffees” food groups in the KNHANES classification system. Food names and product names were then used for keyword-based classification, followed by manual review of ambiguous records to improve classification accuracy and ensure consistency across survey years. Caffeinated beverages were classified into five categories: coffee, tea (including green and other teas), energy drinks, cola-type beverages, and cocoa-based beverages. Non-beverage sources of caffeine were excluded from the analysis (Supplementary Figure S1). The complete list of dish codes and detailed classification criteria is provided in Supplementary Table S1.

### Classification of beverage subtypes

Coffee beverages were subclassified into liquid coffee, instant coffee powder, prepared coffee mixes, and roasted bean or capsule coffee. Tea beverages were classified as liquid, powdered, or infusion-type. Energy drinks, cola-type beverages, and cocoa-based beverages were treated as single subtypes (19). Decaffeinated beverages were identified separately using product descriptions and assigned minimal caffeine content.

### Assignment of caffeine content and estimation of daily intake

Caffeine concentrations (mg/L) were assigned using mean values from national monitoring data published by the Ministry of Food and Drug Safety (20). To enhance temporal comparability, fixed mean concentrations were applied uniformly across all survey cycles. The applied mean caffeine concentrations were: liquid coffee (352.9 mg/L), instant coffee powder (293.5 mg/L), prepared coffee mixes (571.9 mg/L), roasted bean or capsule coffee (522.8 mg/L); liquid tea (105.8 mg/L), powdered tea (115.0 mg/L), infusion-type tea (143.6 mg/L); energy drinks (320.8 mg/L); cola beverages (187.5 mg/L); and cocoa-based beverages (360.9 mg/L). Decaffeinated beverages were assigned a nominal caffeine concentration of 1 mg/L to reflect minimal residual caffeine content.

Caffeine intake per record (mg) was calculated by multiplying beverage volume (L) by the assigned concentration (mg/L). Total daily caffeine intake (mg/day) was obtained by summing intake across all beverages consumed on the recall day (21).

### Statistical analysis

All analyses were performed using the *survey* package in R (version 4.4.2; R Foundation for Statistical Computing, Vienna, Austria). The complex sampling structure of KNHANES was incorporated by applying sampling weights, stratification variables, and primary sampling units in all analyses. To obtain nationally representative estimates for the pooled 2016–2024 period, original annual sampling weights were divided by nine, in accordance with the KNHANES analytic guidelines.

Participant characteristics were summarized as unweighted counts and weighted percentages. Characteristics of caffeine consumers and non-consumers were compared using the Rao–Scott adjusted chi-square test for categorical variables. For variables with missing information, missing values were treated as a separate category to preserve the representativeness of the weighted sample and minimize data loss.

Mean caffeine intake was estimated according to beverage category, sex, and age group. Temporal trends in mean caffeine intake and high caffeine intake were assessed using survey-weighted linear and logistic regression models, respectively, with survey year treated as a continuous variable. High caffeine intake was defined as ≥400 mg/day on the recall day and analyzed as a binary outcome (≥400 vs. <400 mg/day).

Associations between participant characteristics and high caffeine intake were examined using weighted multivariable logistic regression models. Covariates—survey year, sex, age group, body mass index (BMI), socioeconomic status (education level, household income quartile, and occupation) (22), and lifestyle factors (alcohol consumption, smoking status, breakfast frequency, and average sleep duration) (23)—were sequentially added across three models to evaluate the robustness of the associations. Adjusted odds ratios (aORs) and 95% confidence intervals (CIs) were calculated. Statistical significance was defined as a two-sided *P* < 0.05.

## Results

Of the initial 67,019 participants, individuals aged <19 years (*n* = 11,677) and those with missing 24-h dietary recall data (*n* = 6,351) were excluded. Therefore, 48,991 Korean adults aged ≥19 years from the 7–9th Korea National Health and Nutrition Examination Survey (2016–2024) were included in the final analysis. Among them, 31,056 participants (64.5%) were classified as caffeine consumers, while 17,935 (35.5%) were classified as non-consumers (Figure 1).

**Figure 1:**
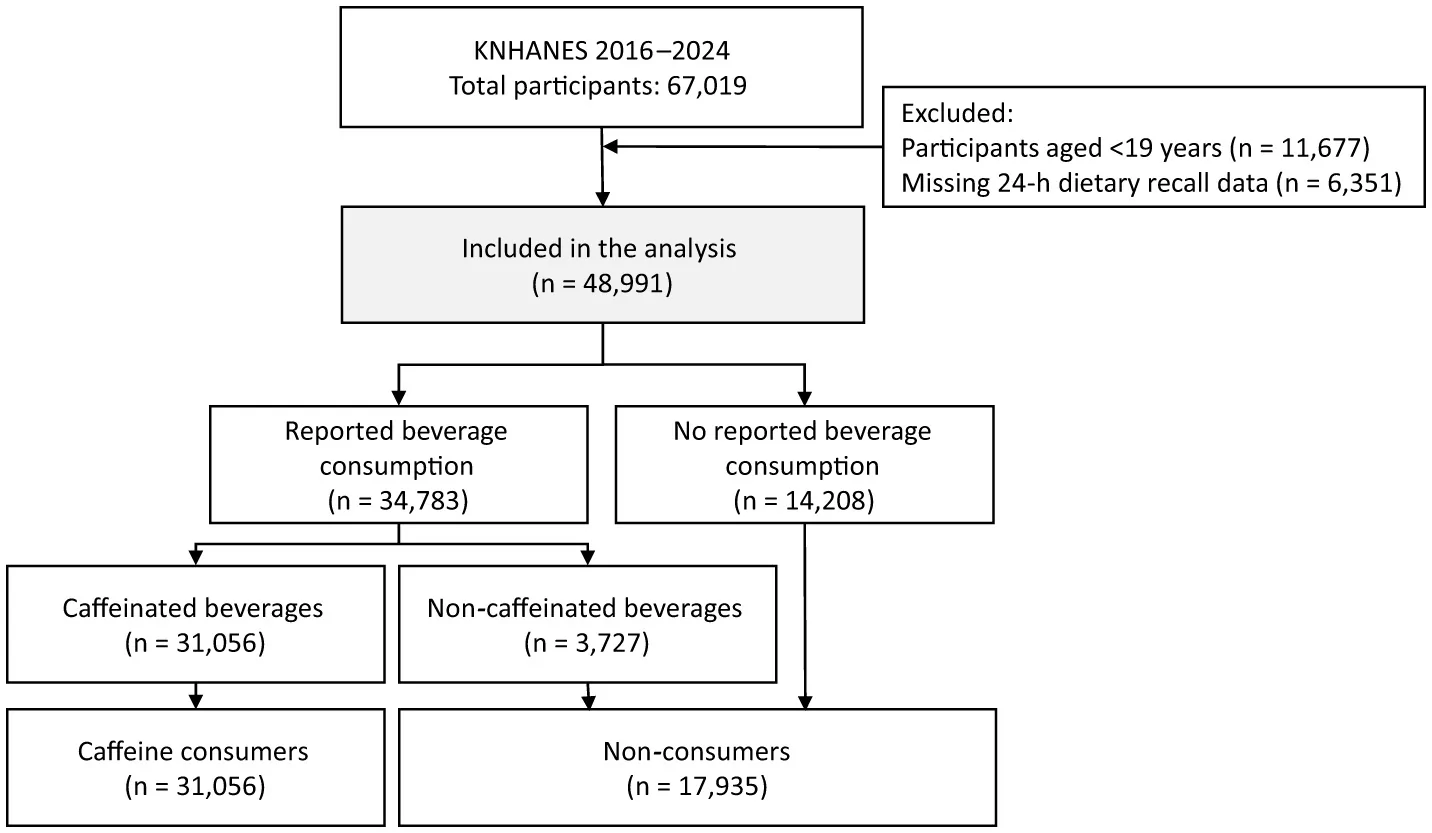
Flow diagram of participant selection from the Korea National Health and Nutrition Examination Survey (KNHANES), 2016–2024. Of the initial 67,019 participants, individuals aged <19 years (*n* = 11,677) and those with missing 24-hour dietary recall data (*n* = 6,351) were excluded, leaving 48,991 adults for the final analysis. Participants were classified according to reported beverage intake and caffeine consumption status. Among all adults, 31,056 were identified as caffeine consumers and 17,935 as non-consumers. Caffeinated beverage subtypes included coffee, cola-type beverages, tea (including green and other teas), energy drinks, and cocoa-based beverages. KNHANES, Korea National Health and Nutrition Examination Survey; RTD, ready-to-drink.

Compared with non-consumers, caffeine consumers were more likely to be male, younger, employed, and have higher levels of education and household income (all *P* < 0.001). The distribution of survey years did not differ significantly between groups (Table 1).

**Table 1:** Characteristics of Korean adults by beverage-based caffeine consumption, KNHANES 2016–2024.

Characteristic	Overall	Caffeine consumers	Non-consumers	*P* value
	48,991 (100.0)	31,056 (64.5)	17,935 (35.5)	
**Survey year**				0.310
2016–2018	16,856 (32.5)	10,811 (32.8)	6,045 (32.1)	
2019–2021	15,549 (33.6)	9,840 (33.4)	5,709 (33.8)	
2022–2024	16,586 (33.9)	10,405 (33.8)	6,181 (34.1)	
**Sex**				<0.001
Male	21,037 (49.7)	14,137 (52.4)	6,900 (45.0)	
Female	27,954 (50.3)	16,919 (47.6)	11,035 (55.0)	
**Age group (years)**				<0.001
19–29	5,700 (16.9)	2,960 (13.7)	2,740 (22.5)	
30–44	10,803 (25.9)	7,537 (28.1)	3,266 (22.1)	
45–64	18,180 (37.1)	12,779 (41.0)	5,401 (29.8)	
≥65	14,308 (20.1)	7,780 (17.2)	6,528 (25.5)	
**Body mass index (kg/m** ^**2**^ **)**				<0.001
Underweight	1,946 (4.3)	1,094 (3.8)	852 (5.4)	
Normal weight	17,610 (36.8)	10,903 (35.8)	6,707 (38.7)	
Overweight	10,666 (22.2)	6,867 (22.7)	3,799 (21.4)	
Obese	16,621 (35.7)	10,888 (37.0)	5,733 (33.2)	
Missing	2,148 (0.9)	1,304 (0.8)	844 (1.3)	
**Education level**				<0.001
≤ Middle school	13,121 (19.7)	7,332 (17.5)	5,789 (24.0)	
High school	14,715 (33.7)	9,438 (33.3)	5,277 (34.5)	
≥College	17,407 (42.4)	11,981 (45.2)	5,426 (37.2)	
Missing	3,748 (4.2)	2,305 (4.1)	1,443 (4.4)	
**Household income (quartile)**				<0.001
Lowest	12,241 (24.3)	7,354 (22.9)	4,887 (26.7)	
Lower-middle	12,222 (24.7)	7,768 (24.8)	4,454 (24.4)	
Upper-middle	12,254 (25.2)	7,959 (25.8)	4,295 (24.1)	
Highest	12,103 (25.5)	7,878 (26.2)	4,225 (24.3)	
Missing	171 (0.3)	97 (0.3)	74 (0.5)	
**Occupation**				<0.001
Employed	26,725 (60.2)	18,418 (65.1)	8,307 (52.2)	
Unemployed	17,704 (33.8)	9,804 (29.2)	7,900 (42.2)	
Missing	4,562 (6.0)	2,834 (5.9)	1,728 (6.0)	

Values are presented as unweighted numbers (N) and weighted percentages (%). Missing values were included as a separate category. P values were obtained using Rao–Scott χ^2^ tests accounting for the complex survey design. KNHANES, Korea National Health and Nutrition Examination Survey.

Overall, mean daily caffeine intake showed a significant linear increase (β per year = 1.66 mg/day; 95% CI, 1.05–2.27; *P* < 0.001). Although intake was consistently higher in men, significant upward trends were observed in both sexes (Figure 2A), with a steeper annual increase in men (β = 2.22 mg/day/year; 95% CI, 1.30–3.15; *P* < 0.001) than in women (β = 1.10 mg/day/year; 95% CI, 0.41–1.79; *P* = 0.002).

**Figure 2:**
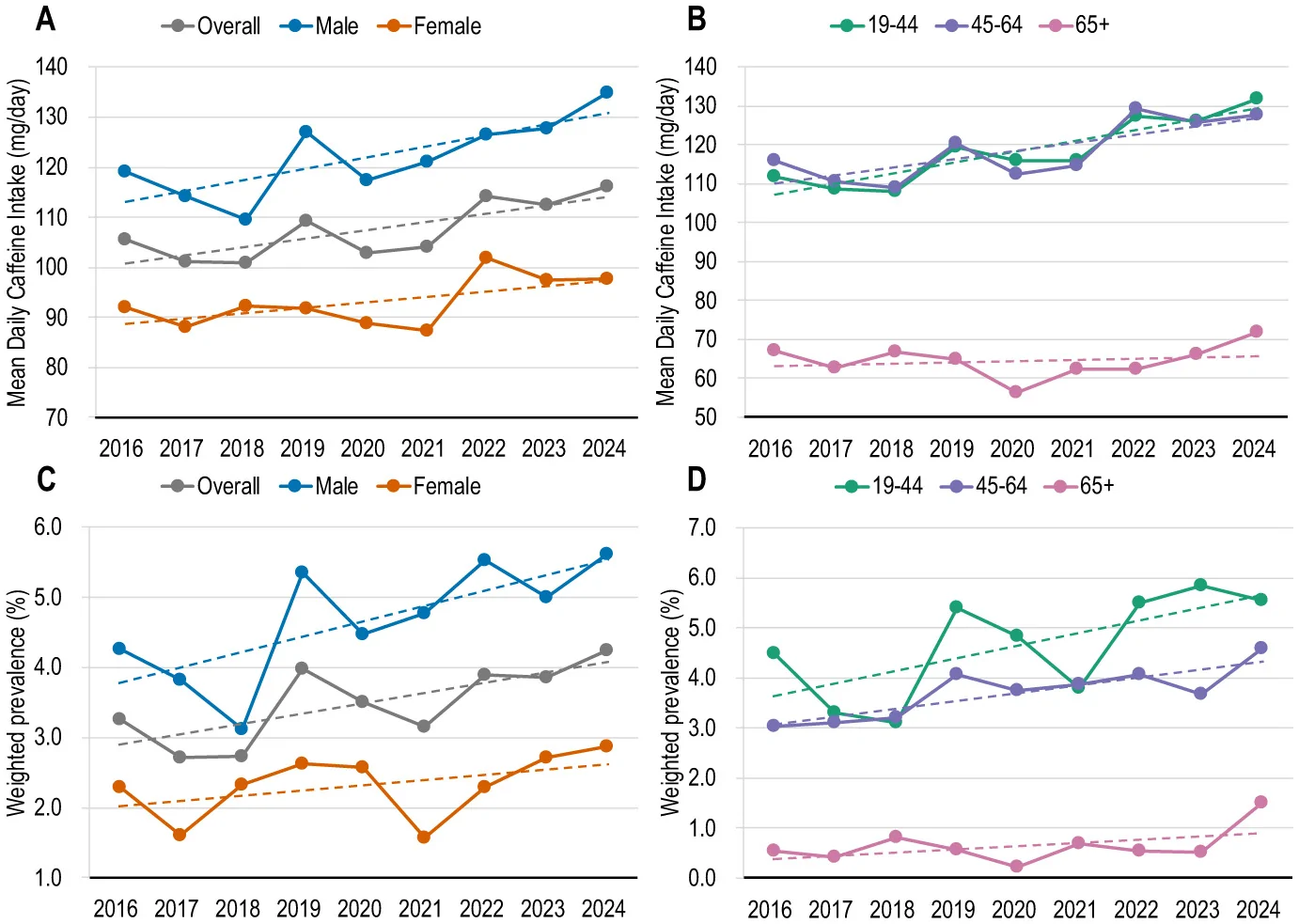
Temporal trends in mean daily caffeine intake and the weighted prevalence of high caffeine intake by sex and age group in Korean adults, KNHANES 2016–2024. **(A)** Mean daily caffeine intake (mg/day) by sex. **(B)** Mean daily caffeine intake (mg/day) by age group. **(C)** Weighted prevalence (%) of high caffeine intake (≥400 mg/day) by sex. **(D)** Weighted prevalence (%) of high caffeine intake (≥400 mg/day) by age group. Dashed lines indicate linear trend lines. All estimates accounted for the complex sampling design and sampling weights of the KNHANES. KNHANES, Korea National Health and Nutrition Examination Survey.

Mean daily caffeine intake increased significantly among adults aged 19–44 years (β = 2.79 mg/day/year; 95% CI, 1.75–3.83; *P* < 0.001) and 45–64 years (β = 2.15 mg/day/year; 95% CI, 1.22–3.08; *P* < 0.001). Conversely, no significant change was observed in mean intake for those aged ≥65 years (β = 0.44 mg/day/year; 95% CI, −0.22–1.09; *P* = 0.193) (Figure 2B).

The weighted prevalence of high caffeine intake (≥400 mg/day) increased over time (Figures 2C, D), while survey-weighted logistic regression analysis showed a significant increasing temporal trend in the odds of high caffeine (OR = 1.05 per year; 95% CI, 1.02–1.07; *P* < 0.001). Sex-stratified analyses showed a significant increase among men (OR = 1.05; 95% CI, 1.02–1.08; *P* = 0.001), whereas the trend among women was not significant (OR = 1.03; 95% CI, 1.00–1.07; *P* = 0.065). Age-stratified analyses demonstrated significant increases among adults aged 19–44 years (OR = 1.06; 95% CI, 1.03–1.09; *P* < 0.001), 45–64 years (OR = 1.05; 95% CI, 1.01–1.08; *P* = 0.015), and ≥65 years (OR = 1.12; 95% CI, 1.01–1.23; *P* = 0.026).

Coffee was the predominant source of caffeine among consumers, while other caffeinated beverages contributed to a smaller proportion of total intake. Among caffeine consumers (*n* = 31,056), coffee was the most commonly consumed beverage (90.8%), followed by cola (13.4%), tea (4.7%), energy drinks (1.6%), and cocoa beverages (0.2%; Figure 3A). As participants could consume more than one beverage type on the same day, summed proportions exceeded 100%. A gradual increase in brewed coffee consumption and a corresponding decline in coffee mix intake were observed, whereas instant and ready-to-drink coffee products remained relatively stable over time (Figure 3B).

**Figure 3:**
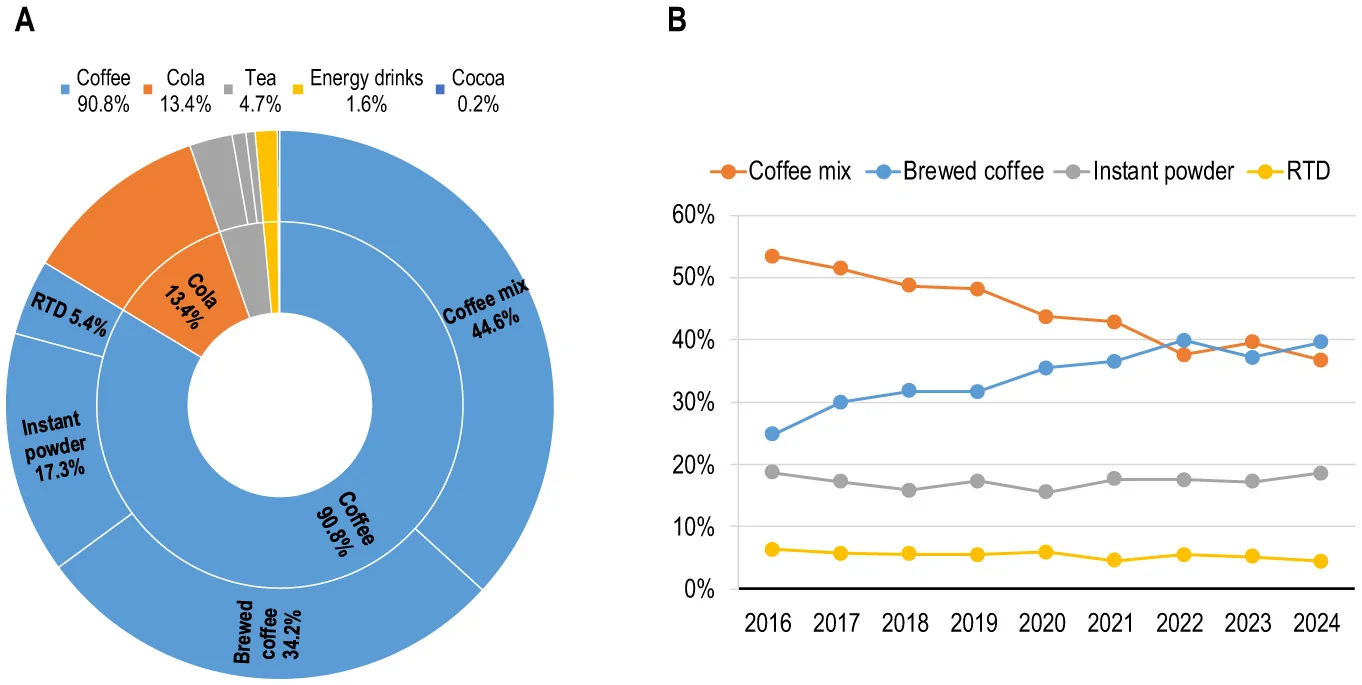
Weighted proportion (%) of caffeine-containing beverage subtypes among caffeine consumers in Korean adults, KNHANES 2016–2024. **(A)** Distribution of caffeinated beverage types among caffeine consumers (*n* = 31,056) identified from 48,991 Korean adults included in the analysis. Coffee was the most commonly consumed caffeinated beverage, followed by cola, tea, energy drinks, and cocoa beverages. Percentages were calculated using complex survey sampling weights and do not sum to 100% because participants could consume more than one type of caffeinated beverage on the same day. **(B)** Temporal trends in the weighted proportion of coffee subtype consumption (coffee mix, brewed coffee, instant coffee, and ready-to-drink products) among caffeine consumers from 2016 to 2024. Weighted proportion was calculated as the ratio of weighted consumers of each coffee subtype to the weighted total number of caffeine consumers for each survey year. All estimates accounted for the complex sampling design and sampling weights of the KNHANES. KNHANES, Korea National Health and Nutrition Examination Survey; RTD, ready-to-drink.

Among men, compared with those aged 19–29 years, men aged 30–44 years showed higher odds of high caffeine intake in crude analyses (OR = 1.53; 95% CI, 1.21–1.93), which remained significant after partial adjustment (Adjusted 1: aOR = 1.53; 95% CI, 1.21–1.93; Adjusted 2: aOR = 1.34; 95% CI, 1.03–1.74); however, it was attenuated and no longer significant after full adjustment (Adjusted 3: aOR = 1.21; 95% CI, 0.93–1.56). No significant associations were observed for men aged 45–64 years across models. Conversely, men aged ≥65 years consistently exhibited significantly lower odds of high caffeine intake across all models (OR = 0.26; 95% CI, 0.19–0.35; fully aOR = 0.37; 95% CI, 0.25–0.53; Table 2).

**Table 2:** Sex-stratified odds ratios for high caffeine intake (≥400 mg/day) by age group in Korean adults, KNHANES 2016–2024.

Subgroup	*N* (%)	Crude	Adjusted 1	Adjusted 2	Adjusted 3
		OR (95% CI)	OR (95% CI)	OR (95% CI)	OR (95% CI)
Male					
19–29	120 (4.5%)	1 (Reference)	1 (Reference)	1 (Reference)	1 (Reference)
30–44	310 (6.8%)	1.53 (1.21–1.93)	1.53 (1.21–1.93)	1.34 (1.03–1.74)	1.21 (0.93–1.56)
45–64	346 (4.9%)	1.09 (0.87–1.36)	1.08 (0.86–1.35)	1.03 (0.80–1.33)	1.01 (0.78–1.31)
≥65	85 (1.2%)	0.26 (0.19–0.35)	0.25 (0.19–0.34)	0.30 (0.22–0.42)	0.37 (0.25–0.53)
Female					
19–29	86 (2.5%)	1 (Reference)	1 (Reference)	1 (Reference)	1 (Reference)
30–44	231 (3.7%)	1.52 (1.15–2.01)	1.52 (1.15–2.01)	1.42 (1.07–1.89)	1.62 (1.21–2.16)
45–64	253 (2.6%)	1.03 (0.79–1.35)	1.02 (0.78–1.34)	1.13 (0.85–1.49)	1.61 (1.19–2.17)
≥65	15 (0.2%)	0.08 (0.04–0.15)	0.08 (0.04–0.15)	0.12 (0.06–0.23)	0.23 (0.12–0.44)

Values are presented as unweighted numbers (N) and weighted percentages (%) of participants with high caffeine intake (≥400 mg/day) within each age group. Odds ratios were estimated using survey-weighted logistic regression models with a quasibinomial family to account for the complex sampling design. The crude model was unadjusted. Adjusted model 1 was adjusted for survey year. Adjusted model 2 was additionally adjusted for body mass index, education level, household income quartile, and occupation. Adjusted model 3 was further adjusted for alcohol consumption, smoking status, breakfast frequency, and average sleep duration. OR, odds ratio; CI, confidence interval; KNHANES, Korea National Health and Nutrition Examination Survey.

Women aged 30–44 years had significantly higher odds of high caffeine intake than those in women aged 19–29 years across all models, including the fully adjusted model (aOR = 1.62; 95% CI, 1.21–2.16). A significant positive association was noted in women aged 45–64 years only after full adjustment (aOR = 1.61; 95% CI, 1.19–2.17). Similar to men of the same age group, women aged ≥65 years had substantially lower odds of high caffeine intake across all models (OR = 0.08; 95% CI, 0.04–0.15; fully aOR = 0.23; 95% CI, 0.12–0.44).

## Discussion

In this study, we provide a comprehensive evaluation of beverage-derived caffeine intake among Korean adults over a 9-year period using nationally representative KNHANES data. By applying a harmonized strategy for beverage identification and caffeine estimation across multiple survey cycles, we identified sustained increases in mean caffeine intake and increasing odds of high caffeine intake, particularly among men and adults of working age.

A methodological strength is the use of both beverage food codes and product information to classify caffeinated beverages across survey cycles. This approach was intended to improve classification consistency despite changes in food coding systems and beverage products over time. The use of nationally representative data and appropriate incorporation of complex survey design further enhance the generalizability of the findings.

Our study demonstrated a sustained increase in both mean caffeine intake and increasing odds of high caffeine intake between 2016 and 2024. Earlier Korean research has also documented associations between caffeine intake and employment, education level, and lifestyle-related behaviors, consistent with our findings among adults of working age (24, 25). This increase was primarily observed among adults aged 19–64 years. These age-specific trends may reflect differences in beverage consumption patterns, possibly driven by greater exposure to specialty coffee culture, longer working hours, and greater reliance on caffeine for alertness and fatigue mitigation among economically active populations (24, 26, 27).

Consistent with previous Korean studies, coffee was the dominant source of caffeine, consumed by over 90% of caffeine consumers (11, 20). The observed shift from coffee mixes toward brewed and specialty coffee is particularly noteworthy, as these products typically contain higher caffeine concentrations and are often consumed in larger portion sizes (28). This transition may partially explain the observed increase in high caffeine intake. These findings emphasize the importance of considering beverage subtype, preparation method, and portion size when assessing caffeine exposure, rather than focusing solely on consumption frequency.

While many domestic and international studies have focused on mean caffeine intake (1, 29), our findings draw attention to changes in the upper tail of the intake distribution. By explicitly examining caffeine intake at or above 400 mg/day in relation to age and sex, this study provides greater alignment with internationally recognized safety thresholds (20, 30).

Higher caffeine intake may contribute to occupational stress, primarily through adenosine receptor antagonism (A1 and A2A subtypes), which inhibits sleep-promoting neural pathways and elevates sympathetic nervous system activity. This blockade promotes catecholamine release and cortisol elevation, potentially creating a vicious cycle of fatigue, dependence, and escalating caffeine consumption, characteristic of working-age populations. Concurrently, caffeine-induced sympathetic activation increases heart rate and blood pressure via β-adrenergic stimulation, while central nervous system effects, including rapid eye movement sleep suppression and heightened arousal, contribute to anxiety symptoms and sleep disturbances (31–34).

Sex-stratified analyses revealed distinct age-related patterns in the odds of high caffeine intake. Higher odds observed in men aged 30–44 years in crude and partially adjusted models were attenuated after full adjustment, suggesting that socioeconomic position and behavioral factors may partially explain the association. Contrarily, adults aged ≥65 years of both sexes consistently exhibited markedly lower odds of high caffeine intake across all models. This pattern likely reflects generational differences in beverage preferences, greater sensitivity to caffeine-related adverse effects, and intentional avoidance related to chronic disease management or sleep quality concerns (35, 36).

Our findings both align with and diverge from observations in Western populations. Studies based on US NHANES data have generally reported stable or modestly declining total caffeine intake over recent decades, despite continued dominance of coffee as the primary source (29, 37). Contrary to US data suggesting sustained intake among older adults, older Korean adults in this study had lower mean caffeine intake, although the odds of high caffeine intake showed a modest upward trend (38). These differences highlight cross-cultural variation in coffee-drinking habits, aging-related dietary behaviors, and social contexts of caffeine use.

Similarly, assessments by the European Food Safety Authority suggest that average adult caffeine intake in European populations remains below 400 mg/day, with high caffeine intake occurring predominantly among younger adults, and evidence of increasing population-level exposure over time is limited (30, 39). Contrarily, our findings indicate increasing odds of high caffeine intake among Korean adults aged 19–64 years, suggesting potential differences in recent consumption trajectories compared with those in Western populations. These discrepancies underscore the importance of country-specific surveillance systems and culturally tailored public health guidance.

This study has some limitations. First, caffeine intake was estimated using a single 24-h dietary recall, which does not capture intra-individual variability and may not fully represent habitual intake. Second, although fixed mean caffeine concentrations were applied to ensure temporal comparability across survey cycles, this approach does not account for brand-specific variations or differences in preparation methods. Third, caffeine from non-beverage sources (e.g., chocolate, medications, or dietary supplements) was not included. Finally, the repeated cross-sectional design of the KNHANES precludes causal inference regarding determinants of increasing caffeine consumption over time.

## Conclusions

In this study, we demonstrate a sustained increase in beverage-derived caffeine intake among Korean adults from 2016 to 2024, with pronounced age- and sex-related disparities. The increasing odds of high caffeine intake among younger and middle-aged adults highlight the need for continued surveillance and targeted public health guidance. Age- and sex-specific guidance, improved labeling practices, and enhanced consumer education may help support informed beverage choices in an evolving beverage environment.

## Data Availability

Publicly available datasets were analyzed in this study. The data presented in this study were derived from the Korea National Health and Nutrition Examination Survey (KNHANES), a publicly available database maintained by the Korea Disease Control and Prevention Agency (KDCA).

## Supplementary material

Supplementary Figure S1 Workflow for classification of caffeinated beverages and estimation of caffeine intake from KNHANES 24-h dietary recall data. Beverage records were identified from KNHANES 24-h dietary recall data using beverage-related food codes. Classification was refined through review of food names and product information, followed by beverage categorization, subtype classification, caffeine concentration assignment, and estimation of daily caffeine intake. Iterative review procedures were applied to improve classification consistency across survey years.
