# Supplementary material for: Beverage-based caffeine intake and high consumption trends among Korean adults, 2016–2024: a nationwide population-based study
Source: Front Nutr. 2026 Oct 19;13:1913422. doi: 10.3389/fnut.2026.1913422 (PMC13724484; doi:10.3389/fnut.2026.1913422)
Supplement: Supplementary file 1 [file Table_1.docx]

**Supplementary Table S1**. Beverage classification criteria, assigned caffeine concentrations, and KNHANES dish codes used to estimate beverage-derived caffeine intake, KNHANES 2016–2024.

| Beverage classification | Assigned caffeine concentration (mg/L) | KNHANES dish codes (No. of codes; last four digits of N_DCODE) | Records, n (%) (N = 57,003) | Consumers, n (%) (N = 31,056) | Representative examples |
| --- | --- | --- | --- | --- | --- |
| Coffee – Prepared coffee mixes | 571.9 | 18: 2011, 2014, 2019, 2026, 2032, 2033, 2034, 2035, 2055, 2082, 2086, 2090, 2091, 2092, 2094, 2095, 2099, 5016 | 26,551 (46.6%) | 15,902 (51.2%) | **Examples:** Coffee mix, vending coffee, instant latte.  **Include:** 커피, 믹스, 라떼, 모카, 카누, 맥심.  **Exclude:** 녹차, 홍차, 코코아, 주스, 허브티.  **Decaffeinated:** 디카페인 |
| Coffee – Roasted bean/capsule | 522.8 | 16: 2013, 2026, 2032, 2033, 2034, 2035, 2044, 2054, 2088, 2090, 2091, 2094, 2095, 2099, 5003, 5016 | 13,291 (23.3%) | 9,698 (31.2%) | **Examples:** Americano, espresso, drip coffee.  **Include:** 아메리카노, 에스프레소, 원두, 드립, 캡슐.  **Exclude:** 녹차, 홍차, 코코아.  **Decaffeinated:** 디카페인 |
| Coffee – Liquid, Ready-to-drink | 352.9 | 11: 2032, 2033, 2034, 2035, 2044, 2081, 2082, 2090, 2091, 2094, 5016 | 1,980 (3.5%) | 1,505 (4.8%) | **Examples:** Georgia, Cantata, Let's Be.  **Include:** 캔커피, 병커피, 조지아, 칸타타, 레쓰비.  **Exclude:** 녹차, 홍차, 코코아.  **Decaffeinated:** 디카페인 |
| Coffee – Instant powder | 293.5 | 17: 2023, 2027, 2028, 2032, 2033, 2034, 2035, 2042, 2044, 2081, 2086, 2090, 2094, 2095, 2099, 5004, 5016 | 9,310 (16.3%) | 6,405 (20.6%) | **Examples:** Instant coffee powder, G7, Maxim Black.  **Include:** 가루, 분말, 인스턴트, 스틱.  **Exclude:** 녹차, 홍차, 코코아.  **Decaffeinated:** 디카페인 |
| Cola-type beverage | 187.5 | 2: 2065, 5013 | 3,635 (6.4%) | 3,257 (10.5%) | **Examples:** Coca-Cola, Pepsi.  **Include:** 콜라, 펩시.  **Exclude:** 맥콜, 콜라겐, 주스. |
| Tea – Infusion-type | 143.6 | 8: 2002, 2011, 2025, 2030, 2044, 2064, 2069, 2090 | 1,208 (2.1%) | 934 (3.0%) | **Examples:** Green tea, black tea, oolong tea.  **Include:** 녹차, 홍차, 우롱차, 말차.  **Exclude:** 율무차, W차, 허브티. |
| Tea – Powdered | 115 | 13: 2002, 2005, 2011, 2014, 2019, 2026, 2032, 2033, 2039, 2044, 2069, 5011, 5012 | 268 (0.5%) | 255 (0.8%) | **Examples:** Matcha powder, tea powder.  **Include:** 녹차, 말차, 홍차.  **Exclude:** 율무차, W차.  **Minimal-caffeine:** 아이스티 |
| Tea – Liquid, Ready-to-drink | 105.8 | 19: 2002, 2010, 2014, 2025, 2026, 2030, 2032, 2033, 2035, 2044, 2068, 2069, 2081, 2090, 2094, 2095, 5011, 5013, 5020 | 188 (0.3%) | 171 (0.6%) | **Examples:** Bottled green tea.  **Include:** 녹차, 홍차, 우롱차.  **Exclude:** 율무차, W차.  **Minimal-caffeine:** 아이스티 |
| Energy drink | 320.8 | 10: 2002, 2025, 2035, 2039, 2044, 2058, 2065, 2066, 5013, 5023 | 490 (0.9%) | 449 (1.4%) | **Examples:** Hot6, Monster, Red Bull, Bacchus.  **Include:** 핫식스, 몬스터, 레드불, 박카스.  **Exclude:** 커피, 코코아. |
| Cocoa-based beverage | 360.9 | 6: 2011, 2027, 2037, 2081, 2083, 2090 | 82 (0.1%) | 73 (0.2%) | **Examples:** Hot cocoa, chocolate drink.  **Include:** 코코아, 초코, 카카오.  **Exclude:** 커피, 모카.  **Decaffeinated:** 카카오닙스차 |

**Table footnotes:**

Example items are representative examples and do not constitute a complete list of products.

Keywords are presented in Korean because KNHANES dietary records are recorded in Korean. Beverage classification was performed using Korean text matching.

Beverage classification was performed using predefined Korean keyword matching and KNHANES dish codes, followed by manual review of ambiguous records before assigning beverage-specific caffeine concentrations.

Assigned caffeine concentrations were based on mean values reported in the caffeine-content database of the Korean Ministry of Food and Drug Safety (MFDS).

KNHANES dish codes (N_DCODE) are five-digit standardized identifiers used in the KNHANES dietary database. Beverage records were initially identified using the last four digits of N_DCODE in combination with Korean food and product names, followed by manual review to ensure mutually exclusive beverage classifications across survey years.

Decaffeinated and minimal-caffeine keywords indicate text-matching terms used to identify beverage records assigned a nominal caffeine concentration of 1 mg/L. These terms were used for analytical classification purposes only and do not necessarily indicate products formally labeled as decaffeinated or caffeine-free.

A participant could contribute multiple beverage records because each beverage item reported in the KNHANES 24-h dietary recall was recorded separately. Record-level and consumer-level counts and percentages were unweighted. Record-level percentages were calculated using 57,003 mutually exclusive beverage records contributed by 31,056 participants with beverage-derived caffeine intake >0 mg/day. Consumer-level percentages were calculated using 31,056 unique caffeine consumers as the denominator. Because participants could consume beverages from multiple categories or subtypes, consumer-level percentages may sum to more than 100%.

Korean keyword definitions: 커피, coffee; 믹스, coffee mix; 라떼, latte; 모카, mocha; 카누, KANU; 맥심, Maxim; 녹차, green tea; 홍차, black tea; 코코아, cocoa; 주스, juice; 허브티, herbal tea; 디카페인, decaffeinated; 아메리카노, Americano; 에스프레소, espresso; 원두, coffee beans; 드립, drip coffee; 캡슐, coffee capsule; 캔커피, canned coffee; 병커피, bottled coffee; 조지아, Georgia; 칸타타, Cantata; 레쓰비, Let’s Be; 가루/분말, powder; 인스턴트, instant; 스틱, stick-type product; 콜라, cola; 펩시, Pepsi; 맥콜, McCol; 콜라겐, collagen; 우롱차, oolong tea; 말차, matcha; 율무차, Job’s tears tea; W차, W Tea; 아이스티, iced tea; 핫식스, HOT6; 몬스터, Monster; 레드불, Red Bull; 박카스, Bacchus; 초코, chocolate; 카카오, cacao; 카카오닙스차, cacao nib tea.
